# Comparison of an interactive with a didactic educational intervention for improving the evidence-based practice knowledge of occupational therapists in the public health sector in South Africa: a randomised controlled trial

**DOI:** 10.1186/1745-6215-15-216

**Published:** 2014-06-10

**Authors:** Helen Buchanan, Nandi Siegfried, Jennifer Jelsma, Carl Lombard

**Affiliations:** 1 Department of Health & Rehabilitation Sciences, F45 Old Groote Schuur Hospital Building, University of Cape Town, Observatory, 7925 Cape Town, South Africa; 2 Current: Department of Psychiatry and Mental Health, J-Block, Groote Schuur Hospital, University of Cape Town, Observatory, 7925 Cape Town, South Africa; 3 During the study: South African Cochrane Centre, Medical Research Council, PO Box 19070 Tygerberg, 7505 Cape Town, South Africa; 4 During the study: School of Public Health and Family Medicine, Falmouth Building, University of Cape Town, Observatory, 7925 Cape Town, South Africa; 5 Biostatistics Unit, Medical Research Council, PO Box 19070 Tygerberg, 7505 Cape Town, South Africa

**Keywords:** Pragmatic trial, Randomised controlled trial, Educational intervention, Occupational therapy, Evidence-based practice, South Africa

## Abstract

**Background:**

Despite efforts to identify effective interventions to implement evidence-based practice (EBP), uncertainty remains. Few existing studies involve occupational therapists or resource-constrained contexts. This study aimed to determine whether an interactive educational intervention (IE) was more effective than a didactic educational intervention (DE) in improving EBP knowledge, attitudes and behaviour at 12 weeks.

**Methods:**

A matched pairs design, randomised controlled trial was conducted in the Western Cape of South Africa. Occupational therapists employed by the Department of Health were randomised using matched-pair stratification by type (clinician or manager) and knowledge score. Allocation to an IE or a DE was by coin-tossing. A self-report questionnaire (measuring objective knowledge and subjective attitudes) and audit checklist (measuring objective behaviour) were completed at baseline and 12 weeks. The primary outcome was EBP knowledge at 12 weeks while secondary outcomes were attitudes and behaviour at 12 weeks. Data collection occurred at participants’ places of employment. Audit raters were blinded, but participants and the provider could not be blinded.

**Results:**

Twenty-one of 28 pairs reported outcomes, but due to incomplete data for two participants, 19 pairs were included in the analysis. There was a median increase of 1.0 points (95% CI = -4.0, 1.0) in the IE for the primary outcome (knowledge) compared with the DE, but this difference was not significant (*P* = 0.098). There were no significant differences on any of the attitude subscale scores. The median 12-week audit score was 8.6 points higher in the IE (95% CI = -7.7, 27.0) but this was not significant (*P* = 0.196). Within-group analyses showed significant increases in knowledge in both groups (IE: T = 4.0, *P* <0.001; DE: T = 12.0, *P* = 0.002) but no significant differences in attitudes or behaviour.

**Conclusions:**

The results suggest that the interventions had similar outcomes at 12 weeks and that the interactive component had little additional effect.

**Trial registration:**

Pan African Controlled Trials Register PACTR201201000346141, registered 31 January 2012. Clinical Trials NCT01512823, registered 1 February 2012. South African National Clinical Trial Register DOH2710093067, registered 27 October 2009. The first participants were randomly assigned on 16 July 2008.

## Background

Evidence-based practice (EBP) is widely advocated as a means of providing effective healthcare interventions, but implementation has proved challenging. A systematic review of occupational therapists’ knowledge, skills, attitudes and behaviour regarding EBP concluded that despite positive intentions and active efforts directed at implementation, occupational therapists in upper-income countries had, at best, moderate EBP knowledge and skills, and application was low [1]. The lack of EBP implementation has been attributed to several practical and conceptual challenges [2]. Barriers such as limited time and skills [3-7] have been widely identified as threats to implementation. If this is the situation in countries where resources are more readily available, it is likely to be worse in developing, or resource-constrained, countries.

A 2004 South African survey of registered occupational therapists (n = 436) similarly found that most respondents had positive perceptions but lacked confidence in EBP skills [1], which 31% attributed to limited knowledge and skills. This finding was affirmed by the low number reporting success finding (46%) and applying evidence (36%). In addition, 25% had received EBP training, suggesting the need for additional training opportunities. Preferences for training were workshops (81.5%) and short in-service training sessions (79.8%) [1].

### Educational interventions to increase the implementation of evidence-based practice

In a cluster randomised trial to determine change in attitudes to EBP in musculoskeletal physiotherapists working in a community trust in the United Kingdom, an interactive evidence-based educational programme (n = 17) was compared with a standard in-service package (n = 13), both of which lasted five hours. Results indicated that confidence in search and appraisal skills increased significantly six months after an EBP educational intervention compared with a standard in-service training programme [8]. As EBP skills were not measured, however, it is unknown whether these attitudinal changes were accompanied by an improvement in skills. Interventions that successfully target attitudes and skills may simultaneously address barriers such as limited time. For example, acquiring search skills to locate pre-appraised sources of evidence may be time-saving. Thus, educational interventions may be one way to increase confidence and skills and thereby improve readiness to implement EBP.

*Cochrane Systematic Reviews* of educational interventions (as defined by Forsetlund *et al.*[9]) to improve professional practice were examined. Although relating to professional practice, these reviews were deemed relevant because competence relies on knowledge as well as other characteristics, such as attitudes and behaviour. The main findings of these systematic reviews are outlined below for each educational intervention.

There was unclear or weak evidence for the effects of tailored interventions that addressed barriers to change [10], teaching critical appraisal skills [11] and printed educational materials compared to other interventions [12]. Educational outreach visits (EOVs) [13] and audit and feedback [14] resulted in small to moderate behaviour changes with those that included EOVs being slightly more effective than audit and feedback alone [13]. EOVs were, however, mainly used to change prescribing behaviour [13]. Multifaceted interventions that included EOVs were slightly more effective than EOVs alone, but the differences were not significant and the reviewers were unable to discount the possibility that the multifaceted interventions may have contributed to the larger differences in non-prescribing behaviour [13].

The review on continuing education sessions reported ‘small to moderate improvements in professional practice,’ but stated that conclusions could not be drawn about their effectiveness compared to other interventions due to the small number of studies involved [9, p14]. Although didactic interventions could change practice, those with an interactive component appeared to be more effective but unlikely to change highly complex behaviours [9]. The effects of educational meetings did not differ significantly from multifaceted interventions and were considered likely to have similar effects to EOVs and audit and feedback [9]. A limitation of the review was the incomplete descriptions of the interventions in the included studies [9], a lack of studies that included occupational therapists, and the predominance of studies based in North America and Europe. The authors advocated further research that compared different types of education [13].

The gaps identified in the systematic review about the effects of continuing education sessions including inadequate descriptions of interventions, difficulties identifying the contribution of specific characteristics of interventions to explain the heterogeneity in the results and the lack of strong evidence to show the superiority of interactive over didactic interventions [9] exposed the need for further research. The demand for additional training to increase confidence and skills in EBP identified in a South African occupational therapy survey [1] provided further impetus for this research. A study was therefore undertaken to test the hypothesis that an interactive educational intervention (IE) was more effective than a didactic intervention (DE) in improving EBP knowledge (primary outcome), attitudes and behaviour (secondary outcomes) at 12 weeks. The alternate hypothesis was that the two interventions were similar.

## Methods

### Trial design

This pragmatic randomised controlled trial (RCT) (hereafter referred to as the Occupational Therapy Evidence-Based Practice (OTEBP) trial) employed a matched pair design. This design controls for potential confounders by grouping participants into pairs based on a blocking variable (in this case, knowledge about EBP) and randomly assigning each participant in the pair to one of two interventions [15].

### Setting

The study was conducted in public health facilities in four district municipalities (Cape Winelands, City of Cape Town, Overberg and West Coast) in the Western Cape. Participants serviced tertiary (n = 3), tuberculosis (n = 1), psychiatric (n = 4) and district/provincially aided hospitals (n = 10), specialised health care facilities (n = 1) and clinics (n = 54).

### Participants

The participants were occupational therapists employed in four district municipalities by the Western Cape Department of Health (DOH) (n = 98). Therapists working at least 20 hours per week were eligible. Managers were included because of their role in putting structures and systems in place to support EBP activities [16] and enabling its successful implementation [17]. For pragmatic reasons, therapists working more than 1½ hours driving time outside Cape Town were excluded. Those leaving the DOH before December 2008 or who knew in advance that they would be on leave, and therefore unable to attend the intervention, were also excluded because it would compromise the outcome data. Recruitment occurred over a nine-month period (November 2007 to July 2008). Continuing professional development points were obtained from the official licensing body, and offered as an incentive to participate. Written informed consent was obtained from each participant.

As the primary outcome instrument (refer to section on outcomes) was modified for the OTEBP trial, there were no data to accurately calculate the required sample size. Therefore, the maximum possible number of participants was recruited so the most precise confidence intervals could be calculated.

### Randomisation

Participants were randomised after completing the baseline questionnaires but before the baseline audit. Baseline Shortened Adapted Fresno Test of Competence in Evidence-based Practice (SAFT) scores were sorted from highest to lowest per stratum, and pairs were matched on same scores first. Where more than two participants had the same score, pairs were matched by facility. For example, if four participants had the same score, those at the same facility were matched so that similar practice profiles were obtained in each group. Where participants were from different facilities, matching was done according to the similarity of the facility. Once all those with same scores were matched, remaining participants were matched by next closest score. Managers were paired according to closest scores. Participants were matched by role (clinician or manager) and baseline knowledge score and individuals in each pair were randomly assigned to receive the IE or DE by coin-tossing. The principal investigator (HB) and research coordinator conducted the coin-tossing procedure together to reduce bias.

### Outcomes

Data were collected at baseline and 12 weeks. The primary outcome, EBP knowledge score at 12 weeks, was measured with the Shortened Adapted Fresno Test of Competence in EBP (SAFT). The SAFT was modified for the study from the Adapted Fresno Test of Competence in EBP (AFT) [18] to reduce respondent burden given its length and item difficulty. It contains three items that test different aspects of EBP knowledge objectively. Responses are graded with a rubric and the total possible score is 30 points. The intra-class correlation coefficient (ICC) was calculated based on data from a pilot study involving 26 participants. Using a two-way random effects model for absolute agreement for single measures (ICC type A,1) [19], the SAFT demonstrated excellent inter-rater reliability (IRR) (ICC = 0.99, 95% CI: 0.97, 1.0) and test-retest reliability (ICC = 0.95, 95% CI: 0.88, 0.98) [1]. The SAFT and grading rubric are available from the first author on request.

Secondary outcomes were EBP attitudes and behaviour at 12 weeks measured by self-report using a modified Knowledge, attitude and behaviour questionnaire (KABQ) [20,21], and an audit checklist developed for the study as an objective measure of behaviour. Modifications to the KABQ were required because it was developed for medical students and thus some of the items and terminology were not relevant for the OTEBP trial participants. Modifications included removing items that were not relevant, changing the terminology to relate to occupational therapy and modifying the rating scales. The six-point rating scale was changed to five-points due to the difficulty differentiating between ‘moderately agree/disagree’ and ‘agree/disagree’, and the continuous rating scale was also changed to a five-point scale as it was unclear how it should be completed [1]. Factor analysis revealed three attitude sub-scales with test-retest reliability varying from poor to fair (positive attitudes: ICC = 0.33; 95% CI = −0.1, −0.7; negative attitudes: ICC = 0.24; 95% CI = −0.2, 0.6; and EBP as useful and an important part of continuing professional development: ICC = 0.42; 95% CI = 0.02, 0.7) [1]. Cronbach’s alpha for each item varied from 0.75 to 0.82.

The audit checklist was developed for the trial to measure the extent to which participants were evaluating the effects of their patient interventions through their daily documentation. The checklist underwent a process of development and was reviewed by an expert panel to evaluate face and content validity and clinical utility. The checklist was based on three existing instruments [9,22,23] and used the International Classification of Functioning, Disability and Health (ICF) checklist [24] as a framework. The final checklist consisted of nine items rated on a dichotomous scale (see Table 1).

**Table 1:** Audit checklist

**Item**	**Yes**	**No**	**Not applicable**
Baseline assessment at impairment-level			
Baseline assessment at activity and participation level			
Goals of intervention			
Evidence base for any occupational therapy intervention performed			
Recording of intervention at impairment, activity or participation level			
Monitoring changes in client’s condition between two or more contacts at impairment level			
Monitoring changes in client’s condition between two or more contacts at activity or participation level			
Re-assessment at impairment level			
Re-assessment at activity or participation level			

Pilot testing of the checklist with 10 records of therapists who did not participate in the trial showed that inter-rater reliability (IRR) was at least moderate for each item (kappa ≥0.60). The checklist score was calculated by adding the total number of ‘yes’ responses. The maximum possible score was nine. Where an item was ‘not applicable’, it was subtracted from nine to determine the total score. Audit scores for each record were converted into percentages and mean scores were calculated for each participant.

### Implementation

#### Data collection

Questionnaires were delivered to participants with a request for their completion within a stipulated time frame. Baseline questionnaires were completed before randomisation. The SAFT (primary outcome) was scored independently by the PI (HB) and a research assistant who was blind to allocation. The grading rubric was used to reduce variation in scoring between raters. Questionnaires were numbered to ensure anonymity. To match 12-week questionnaires and track those that were missing, the PI kept a list of participant names and study numbers. The list was not referred to when grading the SAFT.

For the audit, participants supplied their patient lists for a specific week. Five patients per participant were randomly selected at each data collection point. If five or fewer patients were seen, all listed records were audited. Managers were excluded as they did not carry a patient load. Participants in one municipality (n = 8) who serviced several community health facilities over a large geographical area, faxed their records to a central point. Identifying details were removed to maintain confidentiality.

Audits were conducted by one of two trained research assistants blinded to allocation. The PI attended all audits to ensure the scoring criteria were followed. Scoring discrepancies were discussed and consensus reached. Raters remained consistent for each participant where possible.

#### Interventions

Educational interventions were conducted in August and September 2008. Each type of training was repeated to obtain maximal attendance. To reduce variation between sessions, a consistent observer completed a standard checklist to document the extent to which the provider adhered to the plan for each session. Blinding of participants and the provider was not possible. Although participants were aware of the two interventions, no information was given of their content. The PI was the sole trainer and presented both interventions apart from the IE appraisal session, which was facilitated by an experienced EBP teacher from another department. As the PI was the provider for both interventions, blinding was not possible.

On arrival, participants received a folder of EBP training materials and an ‘evidence package’ containing a minimum of three articles providing evidence for interventions in at least one of their practice areas. The ‘packages’ included evidence-based guidelines, critically appraised papers, systematic reviews or pre-appraised literature considered to be ‘best practice’. Articles were selected according to the hierarchy of evidence with pre-appraised sources being used wherever possible.

The content of the DE and first IE session was similar and consisted of a Microsoft PowerPoint presentation explaining the different steps of EBP. The difference lay in the inclusion of small group discussions and hands-on practical exercises to practice specific skills in the IE. Because the DE did not contain a practical search session, additional slides were included in the presentation to demonstrate specific features for searching specialised and traditional databases. Table 2 provides an outline of the interventions. Details of the content of each intervention are available in Additional file 1.

**Table 2:** Content of the educational interventions

**Interactive educational intervention**	**Didactic educational intervention**
4-hour education session (with notes and ‘evidence packs’)	4-hour education session (with notes and ‘evidence packs’)
2-hour session (1 week later)	
Emailed notes from second session	
Telephone/email follow-up (reminders)	

### Didactic educational intervention

The format for this single four-hour intervention was based on the categorisation outlined in a systematic review [9] and consisted of presentations about EBP and its application. Printed educational materials were included as part of the educational intervention [9]. The focus was on knowledge acquisition rather than skills development or application of concepts. Questions were permitted, but there was no opportunity to practise skills or apply information.

### Interactive educational intervention

The IE was multifaceted, consisting of education sessions, emailed notes and telephone or email reminders [9]. Education sessions were modelled on the intervention provided by McCluskey and Lovarini [25] and included presentations; small group interactive tasks and skills practice, such as developing a research question using the Participant, Intervention, Comparison, Outcome (PICO) format; and online database searching [9]. Two sessions were provided (four and two hours respectively) with approximately six days between them. Session 1 focused on the steps of EBP with practical exercises to develop skills. Session 2 was split into two halves. In the first half, participants wrote down questions and requests for additional input, which were dealt with by the PI. The second half involved small group discussions on evidence-based patient notes, barriers and facilitators to EBP, strategies to address barriers, and actions to strengthen knowledge and use of EBP. Feedback from each small group was recorded and emailed to participants two weeks later. After a further two weeks, participants were telephoned by the research coordinator to check that they had received the emailed notes, establish how they were managing to apply EBP, and gauge their need for additional assistance or information. Participants who could not be reached were emailed.

### Data analysis

Data were analysed using *STATISTICA 8*[26]. An intention-to-treat analysis was not possible because outcome data was incomplete for some participants. Matched pairs with complete outcome data were included in the analysis regardless of whether they had received the intervention or not. For baseline descriptive characteristics, medians and ranges were calculated for numerical variables, and frequencies and proportions for categorical items. Baseline scores for primary and secondary outcomes were determined and median scores and ranges computed.

For the analysis of 12-week outcomes, differences in scores for each matched pair were determined and median scores and ranges calculated. As data for all outcomes were negatively skewed, the Wilcoxon matched-pairs test was used to establish whether median differences in matched pair scores differed significantly from zero. As there were no significant differences between the groups at 12 weeks, the Wilcoxon matched-pairs test was conducted to determine whether any significant within-group changes had occurred. Two-sided significance tests were used throughout. A *P* value of ≤0.05 was considered significant.

### Ethical approval

Ethical approval was received from the Health Sciences Faculty Human Research Ethics Committee, University of Cape Town (REC REF: 259/2006) and the Western Cape Provincial Department of Health (Ref. 19/18/RP37/2008). Confidentiality was protected by using numbers rather than names on audit checklists and questionnaires.

## Results

**Figure 1:**
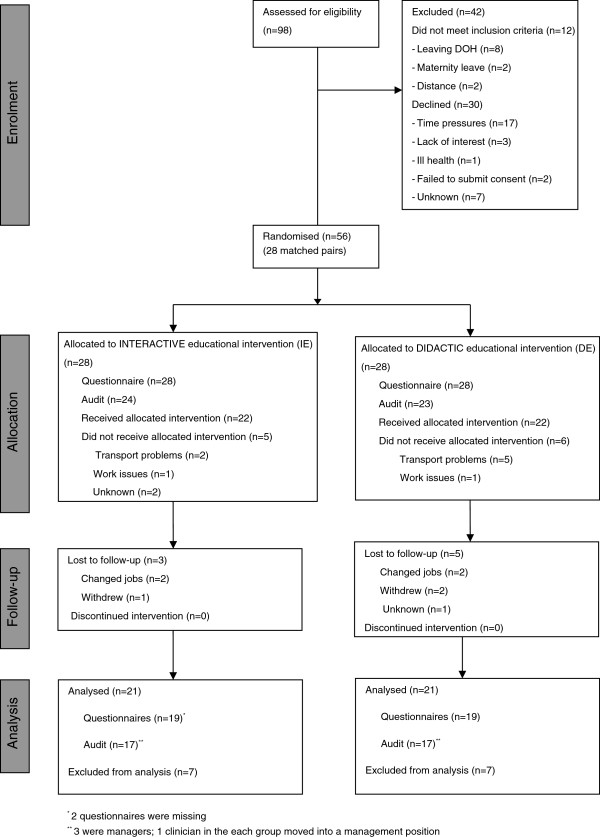
**CONSORT flow diagram for the Occupational Therapy Evidence-Based Practice (OTEBP) Trial.**

The CONSORT flow diagram for the trial is shown in Figure 1. Of the 86 eligible participants, 56 (65.1%) were enrolled to the trial (28 matched pairs). Twelve eligible participants did not meet the inclusion criteria and 30 declined to participate (refer to Figure 1 for reasons for declining). Twenty participants in the IE attended both educational sessions, one attended session one only, and two attended only the second session. In the DE, 22 attended the intervention. Five participants in the IE and six in the DE did not attend the intervention. Group sizes varied from three to 18, according to participants’ availability (refer to Table 3 for details). At 12 weeks, three participants were lost to follow-up in the IE and five in the DE, resulting in 21 matched pairs being included in the final analysis.

**Table 3:** Participant attendance at interventions

**Interactive educational intervention (n = 28)**			**Didactic educational intervention (n = 28)**	
**Session no.**	**Group no.**	**No. (%)**	**Group no.**	**No. (%)**
1	1	18 (64.3)	1	14 (50.0)
	2	3 (10.7)		
	**Total**	**21 (75.0)**		
2	1	13 (46.4)	2	8 (28.6)
	2	9 (32.1)		
	**Total**	**22 (78.6)**		
Total attending full intervention		20 (71.4)	Total	22 (78.6)

### Participant characteristics

Demographic and practice variables (Table 4) were similar for each group apart from age, experience and number of clients per month. Participants were evenly distributed across facilities (Table 5) and most could access all sources of evidence at work or at home (Table 6).

**Table 4:** Baseline characteristics of participants (n = 56)

***Variable***	***Interactive educational intervention (n = 28*** ***)***	***Didactic educational intervention (n = 28)***
	***Median (min to max)***	***Median (min to max)***
Age (years)	28.0 (22.0 to 50.0)	33.0 (22.0 to 56.0)
Experience (years)	5.5 (0.5 to 31.0)	8.5 (0.5 to 34.0)
Number of clients per month^ab^	55.0 (0.0 to 220.0)	35.0 (0.0 to 220.0)
	***n (%)***	***n (%)***
Gender		
Male	2 (7.1)	1 (3.6)
Female	26 (92.9)	27 (96.4)
* Total*	*28 (100.0)*	*28 (100.0)*
Qualification		
Undergraduate	24 (85.7)	24 (85.7)
Postgraduate	4 (14.3)	4 (14.3)
* Total*	*28 (100.0)*	*28 (100.0)*
Level of care		
Primary	5 (17.9)	5 (17.9)
Secondary	9 (32.1)	6 (21.4)
Tertiary	10 (35.7)	12 (42.9)
>1 level	4 (14.3)	5 (17.9)
* Total*	*28 (100.0)*	*28 (100.1)*
Place		
Urban	24 (82.8)	24 (88.9)
Rural	3 (13.8)	4 (11.1)
Both	1 (3.4)	0 (0.0)
* Total*	*28 (100.0)*	*28 (100.0)*

^a^Participant’s estimated the average number of clients seen per month.

^b^Missing data for one interactive educational intervention participant.

**Table 5:** Description of facilities and participant roles (n = 56)

***Type of facility***	**Interactive educational intervention *(n = 28)***				**Didactic educational intervention** ***(n = 28)***			
	***No. of facilities***	***Clinicians no. (%)***	***Managers no. (%)***	***Total no. (%)***	***No. of facilities***	***Clinicians no. (%)***	***Managers no. (%)***	***Total no. (%)***
Tertiary hospital	3	10 (35.7)	0 (0.0)	10 (35.7)	3	12 (42.9)	1 (3.6)	13 (46.4)
District/provincially aided hospital	3	3 (10.7)	0 (0.0)	3 (10.7)	2	2 (7.1)	0 (0.0)	2 (7.1)
Tuberculosis hospital	1	2 (7.1)	0 (0.0)	2 (7.1)	1	1 (3.6)	0 (0.0)	1 (3.6)
Psychiatric hospital	3	2 (7.1)	2 (7.1)	4 (14.3)	3	2 (7.1)	2 (7.1)^a^	4 (14.3)
Clinics	26	4 (14.3)	0 (0.0)	4 (14.3)	32	4 (14.3)	1 (3.6)^b^	5 (17.9)
Specialised health care facilities	1	3 (10.7)	2 (7.1)	5 (17.9)	4	3 (10.7)	0 (0.0)	3 (10.7)
*Total*	*37*	*24 (85.6)*	*4 (14.3)*	*28 (100.0)*	*45*	*24 (85.7)*	*4 (14.3)*	*28 (100.0)*

^a^One clinician who was moving into a management position at another DOH facility was allocated as a manager.

^b^One clinician was appointed as a manager during the study.

**Table 6:** Access to information sources (n = 56)

***Source of evidence-based information***	**Interactive educational intervention *(n = 28)***		**Didactic educational intervention *(n = 28)***	
	***Yes number (%)***	***No number (%)***	***Yes number (%)***	***No number (%)***
Lectures/presentations: Intervention	24 (85.7)	4 (14.3)	27 (96.4)	1 (3.6)
Lectures/presentations: Research	25 (89.3)	3 (10.7)	23 (82.1)	5 (17.9)
Text and reference books	26 (92.9)	2 (7.1)	26 (92.9)	2 (7.1)
Journals	24 (85.7)	4 (14.3)	24 (85.7)	4 (14.3)
Access to academic library	23 (82.1)	5 (17.9)	21 (75.0)	7 (25.0)
Search facilities	24 (85.7)	4 (14.3)	22 (78.6)	6 (21.4)
Connections to world wide web/internet	27 (96.4)	1 (3.6)	25 (89.3)	3 (10.7)
Colleagues working with similar clients	27 (96.4)	1 (3.6)	28 (100.0)	0 (0.0)
Colleagues with expertise	26 (92.9)	2 (7.1)	26 (92.9)	2 (7.1)
Journal club or interest group	22 (78.6)	6 (21.4)	22 (78.6)	6 (21.4)

### Baseline and 12-week scores

Baseline and 12-week scores for primary and secondary outcomes and the median differences in matched pair scores at 12 weeks are shown in Table 7. Scores were similar across groups at baseline. At baseline, 258 records were audited at 15 facilities with a mean of five records for most participants (46/49, 93.9%). Two participants had three records audited and one had four - either because they had not seen five different patients in the selected week or the record was unavailable as it was either in the doctor’s office or being used by another member of the multidisciplinary team at the time. While changes to records could have been made prior to the audit, there was no evidence of this. Audit scores were generally low.

**Table 7:** Baseline and 12-week scores with median differences in 12-week matched pair scores

***Domain***	***Item (possible score)***	**Interactive educational intervention (IE)**		**Didactic educational intervention (DE)**		***Median difference in matched pairs***^***a***^ ***(95% CI)***	***P value for Wilcoxon matched-pairs test***
		***Baseline***	***12 weeks***	***Baseline***	***12 weeks***		
		***Median (min to max)***	***Median (min to max)***	***Median (min to max)***	***Median (min to max)***		
		*(n = 28)*	*(n = 19)*	*(n = 28)*	*(n = 19)*	*n = 19*	
Knowledge	*SAFT score (30)*	*14.0 (2.0 to 23.0)*	*21.0 (2.0 to 25.0)*	*14.0 (1.0 to 23.0)*	*19.0 (9.0 to 24.0)*	1.0 (−4.0, 1.0)	0.098
Attitudes	Negative attitudes to EBP (25)	12.0 (9.0 to 18.00)	11.0 (9.0 to 18.0)^b^	11.0 (9.0 to 16.0)	12.0 (5.0 to 16.0)	0.0 (−2.0, 4.0)	0.728
	Positive attitudes to EBP (15)	12.5 (9.0 to 15.0)	12.5 (7.0 to 15.0)^b^	12.5 (9.0 to 15.0)	13.0 (9.0 to 15.0)	−1.0^a^ (−3.0, 1.0)	0.187
	EBP is useful and important (10)	8.0 (6.0 to 10.0)	8.0 (4.0 to 10.0)^b^	8.0 (2.0 to 10.0)	8.0 (6.0 to 10.0)	0.0 (−2.0, 0.0)	0.064
		*IE (n = 24)*	*IE (n = 17)*	*DE (n = 24)*	*DE (n = 17)*	*n = 17*	
Behaviour	Audit (%)	42.9 (13.7 to 60.0)	45.5 (20.0 to 69.4)	36.3 (14.7 to 76.0)	*39.7 (23.0 to 68.0)*	8.6 (−7.7; 27.0)	0.196

^a^A negative value indicates that the DE scored higher than the IE.

^b^Missing data for one participant.

EBP, evidence-based practice; SAFT, Shortened Adapted Fresno Test of Competence in Evidence-based Practice.

Of the 42 participants (21 matched pairs) who completed the trial, 19 matched pairs completed the questionnaire and 17 completed the audit. The median time between completion was 13.0 weeks (min to max = 10.0 to 22.0 weeks) for the IE and 14.0 weeks (min to max = 12.0 to 17.0 weeks) for the DE. Responsiveness, calculated using baseline and 12-week data from OTEBP trial completers, was large (*d* = 0.92) for the SAFT and small (*d* = 0.3) for the audit checklist [1]. Of the 35 participants whose records were audited at 12 weeks (one clinician moved into a management position during the study), 23 (65.7%) had the same rater for both data collection points. Analysis of matched-pair scores revealed no significant differences for primary or secondary outcomes (see Table 7). Within-group analyses showed significant increases in knowledge in both groups (see Table 8), but there were no differences in the remaining outcomes.

**Table 8:** Within-group changes from baseline to 12 weeks

***Outcome***	***Instrument***	***Group***	***n***	***Baseline***	***12 weeks***	***Wilcoxon matched-pairs test***	
				***Median (min to max)***	***Median (min to max)***	***T value***	***P value***
Knowledge	SAFT	IE	23	14.0 (2.0 to 23.0)	21.0 (2.0 to 25.0)	4.0	**<0.001**
		DE	17	14.0 (1.0 to 23.0)	19.0 (9.0 to 24.0)	12.0	**0.002**
Attitudes	KABQ - negative attitudes to EBP	IE	18	12.0 (9.0 to 18.00)	11.0 (9.0 to 18.0)^a^	75.0	0.647
		DE	17	11.0 (9.0 to 16.0)	12.0 (5.0 to 16.0)	68.5	0.705
	KABQ - positive attitudes to EBP	IE	15	12.5 (9.0 to 15.0)	12.5 (7.0 to 15.0)^a^	53.0	0.691
		DE	14	12.5 (9.0 to 15.0)	13.0 (9.0 to 15.0)	26.5	0.103
	KABQ - EBP is useful and important	IE	14	8.0 (6.0 to 10.0)	8.0 (4.0 to 10.0)^a^	48.0	0.778
		DE	8	36.3 (14.7 to 76.0)	*39.7 (23.0 to 68.0)*	15.0	0.674
Behaviour	Audit	IE	22	41.4 (13.7 to 60.0)	44.7 (20.0 to 76.0)	87.0	0.200
		DE	18	36.3 (14.7 to 76.0)	*39.7 (23.0 to 68.0)*	58.0	0.231

^a^Missing data for one participant.

DE, didactic educational intervention; EBP, evidence-based practice; IE, interactive educational intervention; KABQ, Knowledge, attitude and behaviour questionnaire; SAFT, Shortened Adapted Fresno Test of Competence in Evidence-based Practice.

## Discussion

Despite an increase in median SAFT and audit scores in both groups at 12 weeks, the lack of significance in between-group differences suggests that the interventions had similar effects. A possible explanation for not finding a significant difference in the IE may be the high number in this group who did not attend the full intervention (20 of 28 participants attended both sessions). This may have lessened the impact on knowledge and behaviour resulting in participants achieving lower median change scores than expected. The greater, albeit not significant, improvement in knowledge and behaviour in the IE supports Forsetlund *et al’s*[9] systematic review conclusion that educational interventions tend to be more effective when an interactive component is included. However, the OTEBP trial suggests that where baseline knowledge is low, any mode of education may make a difference. The point difference of 1.0 for knowledge is possibly indicative of a real difference that was not detected, but uncertainty remains given the lack of precision indicated by the confidence interval, which includes both benefit and appreciable harm.

The lack of significant changes in attitudes in either group at 12 weeks may indicate that neither intervention influenced attitudes or it may reflect the poor reliability of the attitude sub-scales in the KABQ. According to Smith *et al.*[27], changing behaviour at the individual level requires the person to identify the need for change as well as the motivation to move from the stage of contemplation to action. They concluded that not everyone involved in a programme aimed at changing practice will actually implement the necessary changes [27]. The fact that both interventions were relatively short in duration resulted in an emphasis on knowledge and skills rather than focussing on strategies targeted at positive attitudinal change.

The low median audit scores at baseline and 12-weeks showed that participants were not documenting information related to patient interventions sufficiently to be able to evaluate the effects of their interventions. Incomplete documentation was similarly identified in an audit of occupational therapy stroke records at an academic hospital in South Africa [28]. It was disappointing that there were no significant improvements in audit scores at 12 weeks particularly considering that the second IE session contained a discussion on evidence-based record-keeping. Studies involving occupational therapists [25] and public health physicians [29] similarly found little difference in EBP behaviour after an educational intervention. The researchers concluded that while no behaviour change was seen, changes in knowledge and attitudes may, in fact, precede changes in behaviour [29], and that changes are needed at both individual and organisational levels for EBP to be implemented successfully [25]. By contrast, a multifaceted evidence-based medicine (EBM) intervention with 47 doctors in a department of medicine in the UK, was effective in improving practice [30]. Interestingly, none of the doctors involved in the study had prior EBM training, and yet after seven hours of training, significantly more of their patients received interventions shown to be beneficial in RCTs [30].

### Generalisability

The findings of this trial are specific to occupational therapists working in the public health sector in the Western Cape and other urban areas in South Africa. The extent to which the findings are applicable to occupational therapists working in other sectors, such as private practice, or other government departments, such as the Department of Education, is uncertain. The fact that participants were self-selected due to the ethical requirement of informed consent may have positively biased the effects of the intervention as participants may have been more motivated to learn about and apply EBP than those who declined. Findings may therefore not be generalisable to all occupational therapists but rather to those interested in learning more about EBP.

### Strengths and limitations

Controlling for possible allocation bias by balancing knowledge levels across groups was a strength of the study. While coin-tossing was a quick, feasible method of randomisation at the time, it is not a satisfactory method due to the possibility of manipulating the outcome according to the technique used and thereby introducing bias [31]. In response to the criticism that previous educational evaluation studies relied on self-reports that can overestimate effects [32], the OTEBP trial included objective measures of knowledge and behaviour, thereby strengthening the validity of the results. Furthermore, the study sought to address the failure of researchers to provide detailed descriptions of interventions [9].

The study would have been strengthened had a blinded assessor, rather than the PI, scored the SAFT as this may have raised questions about bias. The low test-retest reliability of the KABQ attitude subscales and audit checklist may have contributed to the lack of significant findings. As there were no other instruments available for measuring EBP attitudes or behaviour at the time, and considering they measured secondary outcomes, a decision was made to continue with the instruments despite this limitation. The findings related to the secondary outcomes should, therefore, be treated with caution. At the end of the trial, two 12-week questionnaires were missing for the DE, but the data could not be re-collected as the 12-week measurement period had passed. Missing data were not imputed due to the possibility of introducing uncertainty and bias [33,34]. Therefore, only matched pairs with complete outcome data were included in the analysis whether they had received the intervention or not. The high proportion of missing data may have led to the non-significant findings and is a further reason for exercising caution when interpreting the findings. As the number lost to follow-up and reasons for non-completion were similar in each group, it is unlikely that participants left the study as a result of the interventions.

Complete outcome data were available for 19 matched pairs for the primary and all secondary outcomes, apart from audit data for which there were 17 matched pairs. However, this represented data from only two-thirds of the included participants indicating a high level of attrition. Despite the high level of engagement between researcher and participants, clinical priorities and departmental obligations prevented many participants from remaining fully active in the trial for the entire period. The fact that one-third of the eligible participants declined to be enrolled in the trial reflects on the acceptability of the intervention and the generalisability of the reported results to this target group. The trial sample size was restricted to the number of available occupational therapists in the public sector. Increasing the sample to include therapists working in other sectors, such as education or private practice, would have increased precision, but it would also have increased the complexity of the trial and was not feasible. Further limitations include the reasonably high loss to follow-up resulting from incomplete data, lack of intention-to-treat analysis and baseline differences, all of which may introduce a high risk of bias. The results should be treated with caution and may not be a true reflection of the benefits or not of the interactive intervention.

Contamination was difficult to prevent due to the strong possibility that participants may have had contact either socially or at other work- or profession-related activities. As this would be likely to occur in ‘usual’ practice, no attempt was made to prevent exchange of information among participants. This may have resulted in improvements in knowledge in both groups, which would underestimate an effect.

## Conclusions

The OTEBP study showed no differences in the trial outcomes at 12 weeks. Participating in either intervention produced a substantial increase in knowledge at 12 weeks. Thus, it seems that the interactive component had very little additional effect.

## Abbreviations

AFT: Adapted Fresno Test of Competence in Evidence-based Practice; DE: didactic educational intervention; DOH: Department of Health; EBP: evidence-based practice; EOVs: educational outreach visits; ICC: intra-class correlation coefficient; ICF: International Classification of Functioning, Disability and Health; IE: interactive educational intervention; IRR: inter-rater reliability; KABQ: knowledge, attitude and behaviour questionnaire; OTEBP trial: Occupational Therapy Evidence-Based Practice Trial; PI: principal investigator; PICO: participant, intervention, comparison, outcome; RCT: randomised controlled trial; SAFT: Shortened Adapted Fresno Test of Competence in Evidence-based Practice.

## Competing interests

The study was conducted as part of HB’s doctoral thesis. No competing interests were declared by the authors.

## Authors’ contributions

HB, NS and JJ conceptualised this trial, which was conducted as part of HB’s doctoral degree. HB applied for, obtained and managed the funding for the study. HB was the principal investigator who managed and conducted all aspects of the trial under the supervision of NS and JJ. CL advised on the trial design and statistical analysis. HB drafted the paper and NS, JJ and CL commented on draft versions. All authors read and approved the final manuscript.

## Supplementary Material

Additional file 1 Content of the educational interventions. Click here for file
