# Supplementary material for: Comparison of an interactive with a didactic educational intervention for improving the evidence-based practice knowledge of occupational therapists in the public health sector in South Africa: a randomised controlled trial
Source: Trials. 2014 Jun 10;15:216. doi: 10.1186/1745-6215-15-216 (PMC4061109; doi:10.1186/1745-6215-15-216)
Supplement: Additional file 1 — Content of the educational interventions. [file 1745-6215-15-216-S1.docx]

# Content of the educational interventions

## Interactive educational intervention (IE)

| **Session 1 outline (4 hours)**  ***Introduction:***  Announcements about OT EBP study  Introductions - participants introduce themselves (name, place and area of work, what they know re EBP & what they hope to learn from session)  Training outline and programme  ***What is EBP?***  Misconceptions  Definitions: Participants identify main elements in the definitions by Sackett *et al* (2005) and Dawes *et al* (Jackson *et al.*, 2006)  ***Why should we use EBP?***  Large group discussion  ***The 5 steps of EBP***  **Step 1: Formulating questions based on clinical scenarios** – example of scenario and different types of questions that could be asked.  **Exercise 1 (small groups):** Identify at least one possible question you would like to answer in your area of practice (small group). Write it as a question you could use in a search. (PI moves around groups to provide assistance where necessary). Feedback from groups  **Step 2: Finding research evidence to answer the question**  What is evidence?  What type of evidence is best?  Primary and secondary research  How to search for evidence  **Exercise 2:** Write a search strategy for the question developed in exercise 1. Discuss with your group and get feedback. Facilitator leads discussion to refine questions and assist groups to identify possible search terms  Doing an efficient search  Where to look for evidence – specialised and traditional databases, guideline sites.  Facilitator demonstrates resources available from **specialised** internet databases:   - Cochrane library – evidence provided; home page; different libraries; access though SA Cochrane Centre - OTSeeker – evidence provided; quality rating scale; home page; how to do a search using drop down menus and key words; how to open article; where to find abstract; how to get full text - PEDro – as for OTSeeker - OTCATs – home page; how to get list of CATs; quality   Facilitator demonstrates resources available **in traditional databases:**   - Pubmed - Clinical queries 🡪 specialised search filters to make searching for answers to clinical questions more efficient – can specify type of question (therapy, diagnosis, prognosis or harm) or search for systematic reviews of primary studies only - CINAHL   **Exercise 3:** Participants practise searching different databases for evidence to answer their PICO question  **BREAK**  **Step 3: How to appraise an article**  Discussion (large group) of article on Depression & exercise using GATE diagram & article (The World Bank Group, 2010c)  **Step 4: Applying evidence & evaluating practice**  Small group discussion: What do you need to consider when deciding whether/how to apply evidence?  ***Where to from here?***  Explain content of folders and evidence ‘packages’.  Task before next session: go through evidence ‘packages’. At next session application of evidence in packages, record-keeping and any other issues identified by participants will be discussed. |
| --- |
| **Session 2 outline (2 hours)**  ***Outstanding issues from last session***  Reflect on what was covered in last session and identify any areas in which you would like more input/information. Write these on the yellow pieces of paper and pass to the front.  The facilitator sorts through questions and discusses each with group in the first half of the session.  **BREAK**  ***Occupational therapy records***  Small group discussion:  How should evidence be documented in OT notes? When should it be included and when not?  What do you think OT records should look like if they are evidence-based?  Extract principles from each group – record on flip chart. Facilitator - outline from article in Australian OT journal  ***What barriers and facilitators are there in your work context that either make if difficult for you to use EBP or facilitate this?***  Small group discussion:  What are the barriers that could make it difficult for you to use EBP?  What facilitators/enablers exist in your work environment that support EBP? Identify resources in your context you could access that would assist you to use EBP, e.g. CATs and CAPs, systematic reviews, journal clubs  Think of ways in which barriers could be overcome/minimised.  Feedback from each group – record on flip chart.  ***What can I do from here to either start using EBP or to strengthen my use of EBP? What changes will I need to do this? What supports can I draw on to help me succeed?***  Small group discussion and make a list of suggestions of how you could continue developing your skills in and use of EBP  Feedback from groups – record on flip chart. Give handout with suggestions after discussion.  ***Closure***  Summarise steps needed for EBP and outline strategies participants identified to help them use EBP  Encourage participants to contact facilitator for further help  Completion of evaluation forms |

## Didactic educational intervention (DE)

| **Outline (4 hours)**  ***Introduction:***  Announcements about EBP study  Introductions - Participants introduce themselves (name, place and area of work, what they know re EBP & why they think EBP is important to their work)  Training outline and programme  ***What is EBP?***  Misconceptions  Definitions: Identify main elements in the definitions by Sackett *et al* and Dawes *et al*  ***Why should we use EBP?***  ***The 5 steps of EBP***  **Step 1: Formulating questions based on clinical scenarios** – example of scenario and different types of questions that could be asked  **Step 2: Finding research evidence to answer the question**  What is evidence?  What type of evidence is best?  Primary and secondary research  How to search for evidence  Doing an efficient search  Where to look for evidence – specialised and traditional databases, guideline sites  Powerpoint slides used to show different **specialised** and **traditional** databases:   - Cochrane library – evidence provided; home page; different libraries; access though SA Cochrane Centre - OTSeeker – evidence provided; rating to help you assess quality; home page; how to do a search using drop down menus etc; how to open article; where to find abstract; how to get full text - PEDro – as for OTSeeker - OTCATs – home page; how to get list of CATs; quality - Pubmed - Clinical queries 🡪 has specialised search filters to make searching for answers to clinical questions more efficient – can specify type of question (therapy, diagnosis, prognosis or harm) or search for systematic reviews of primary studies only - CINAHL   **BREAK**  ***Step 3: How to appraise an article***  ***Step 4 & 5:: Applying evidence & evaluating practice***  ***Evidence-based record-keeping***  Mention briefly the information that should be recorded in patient records  ***Where to from here?***  Explain what is included in folders and evidence packages  Give suggestions about how participants could become evidence-based practitioners    ***Closure & evaluation***  Questions  Completion of evaluation forms |
| --- |
